# Unveiling the Challenges of Gender-Based Violence NGOs in South Africa

**DOI:** 10.1177/10778012251348425

**Published:** 2025-06-11

**Authors:** Jemma Kieser, Carren G. Duffy

**Affiliations:** 1 School of Management Studies, 37716 University of Cape Town, Cape Town, South Africa

**Keywords:** gender-based violence, South Africa, decision-making, funder influence, challenges

## Abstract

Despite heightened awareness and activism, South Africa grapples with alarming rates of gender-based violence (GBV). This exploratory qualitative research investigated the trends, practices, and drivers of decision-making in the GBV domain to understand the complexities of addressing this pervasive issue. Thematic analysis of the interviews with staff from non-governmental organizations (NGOs) revealed that South African NGOs interviewed predominantly engage in secondary and tertiary strategies to address GBV, with little focus on primary intervention. In addition, NGOs’ decision-making is influenced greatly by funder requirements. This paper highlights problematic practices in the GBV NGO context in South Africa and recommendations for improvements.

## Introduction

Gender-based violence (GBV) remains a dire and pervasive reality in South Africa, and the government has crafted one of the most exhaustive definitions of GBV to include physical, emotional, verbal, psychological, and economic abuse, as well as intimidation, harassment, stalking, and damage to property, among others. It has also passed progressive legislation to augment criminal legislation, e.g., the Criminal and Related Matters Amendment Act 12 of 2021, the Criminal Law (Sexual Offences and Related Matters) Amendment Act 13 of 2021, and the Domestic Violence Amendment Act 14 of 2021, and formulated the National Strategic Plan on Gender-based Violence and Femicide 2020–2030 (Republic of South Africa [RSA], 2020), as part of efforts to curb this widespread phenomenon. Yet, despite heightened awareness, activism, and intervention efforts, the alarming prevalence of GBV shows no signs of abating.

According to The Africa Health Organisation (2021), approximately 51% of women in South Africa reported experiencing GBV, often at the hand of their intimate partner, while 76% of men admitted to having committed GBV at some point in their life. According to the South African Police Service's Strategic Management Report (2023) for the period 2022–2023, 54 040 cases of sexual offenses, including rape, were reported—an average of 148 cases per day. Femicide claimed the lives of 3,422 women during the same period. Moreover, these alarming statistics likely understate the true extent of the problem, as many cases of GBV remain unreported, and there is an alarming attrition of cases from reporting to a verdict handed down in court (RSA, 2020).

The persistent prevalence of GBV suggests that there may be underlying challenges hindering the operations and effectiveness of the interventions of the many NGOs focused on addressing GBV. This research, therefore, sought to investigate the trends, practices, and drivers of decision-making in this sphere, with a focus on understanding the complexities involved in addressing this pervasive issue, including the actions of role players in the design and execution of initiatives. The study was aimed at answering the following research questions: What types of GBV interventions are offered in South Africa?; How do funders and funding requirements affect the operations of South African GBV NGOs, including the design, implementation, and evaluation of interventions?; How does scientific evidence-based literature influence GBV NGOs’ intervention design and implementation?; and How do South African GBV NGOs experience collaboration, learning, and information sharing?

### Intervention Approaches and Design

The South African government's (2020) National Strategic Plan on GBV is anchored in six pillars: (1) accountability, co-ordination, and leadership; (2) prevention and rebuilding social cohesion; (3) justice, safety, and protection; (4) response, care, support, and healing; (5) economic power; and (6) research and information management. The numerous strategies used in combating GBV and its effects are broadly categorized into three levels: primary, secondary, and tertiary.

Primary initiatives are strategies aimed at preventing the occurrence of violence by addressing its root causes. Typical initiatives include educational outreach and embedding healthy social norms at societal and situational levels (Dworkin & Barker, 2019; see also Cooper et al., 2013). The rationale behind primary interventions is that eliminating GBV is a matter of social justice at the collective level. If the goal is to end GBV, then men (and boys), as the primary perpetrators of violence against women, should be stakeholders and participants in these interventions (Dworkin & Barker, 2019).

Research by Gevers et al. (2013) identified common strategies for primary prevention interventions, including (a) promoting gender equality and challenging hegemonic masculinities—also referred to as gender-transformative interventions—that address sociocultural norms and attitudes that tolerate or accommodate violence; (b) reducing substance abuse; (c) fostering relationship-building skills; and (d) enhancing conflict resolution and communication skills.

Researchers propose that gender-transformative interventions play a crucial role in promoting gender equality. This approach involves challenging men's rigid gender role beliefs and examining how community, social-cultural, and institutional factors shape individual attitudes and beliefs (Dworkin & Barker, 2019; see also Casey et al., 2013). The goal is to engage men (and boys) in fostering gender-equitable masculinities and encouraging them to actively contribute to the prevention of GBV (Dworkin & Barker, 2019). A review by Barker et al. (2010) found that gender-transformative approaches can be effective, particularly when incorporating group education, mass media campaigns, and community outreach and mobilization (Barker et al., 2010; see also Carlson et al., 2015).

Primary prevention interventions in South Africa aim to foster gender-equitable and non-violent attitudes and behaviors among men *through MenEngage, One Man Can, MenCare+* programs, and awareness and advocacy campaigns. Evaluations have shown positive impacts from these interventions (Gibbs et al., 2020; see also Jewkes et al., 2008; Skevington et al., 2012). Casey et al. (2013) highlight the *MenCare* global program as effective in initiating and engaging men by centering discussions on topics relevant to them, such as fatherhood, effective parenting, and reflections on positive, healthy masculinity. Similarly, Casey et al. (2017) found that GBV prevention strategies that tailor messaging to men's specific concerns were rated most highly by participants who had previously taken part in such interventions.

While primary-level strategies are undeniably a crucial step in the ultimate eradication of GBV, the process of altering deep-rooted societal beliefs is slow, which is why these interventions should be complemented with secondary and tertiary initiatives.

Secondary and tertiary GBV interventions are similar in that both focus on supporting survivors. However, they differ slightly in their overarching aim. Secondary interventions seek to detect violence early and prevent its escalation or recurrence (Kirk et al., 2017), by providing support and resources. Interventions available offer women a wide range of aid, such as counseling, legal and economic empowerment, legal advice and advocacy, workshops and training, and shelter services. These interventions aim to empower women to remove themselves from adverse situations.

Tertiary interventions aim to mitigate the adverse consequences of violence (Kirk et al., 2017) by focusing on the long-term recovery, rehabilitation, and reintegration of survivors. Survivors of GBV often suffer crippling physical, mental, and sexual health outcomes due to posttraumatic stress, depression, and anxiety (O'Brien & Macy, 2016). In some cases, survivors may find it challenging to participate in physical and psychological treatments effectively. This, coupled with a lack of economic resources, often results in women remaining in situations where violence is likely to reoccur (Kim et al., 2007). Tertiary responses to GBV include ensuring greater access to justice for survivors and providing a broad range of support options to the women and children who have been affected by violence.

A systematic review by Kirk et al. (2017) investigated the success of secondary and tertiary prevention strategies for GBV in low- and low-middle-income countries and found that secondary strategies that target substance abuse (mainly alcohol), both among perpetrators and survivors, are successful in reducing GBV. Psychotherapy as a tertiary strategy, which is aimed at assisting survivors of GBV, was also found to be valuable (Kirk et al., 2017).

Various secondary and tertiary GBV interventions operate in South Africa, as the country's effort has been focused predominantly on this response (Safer Spaces, 2020). These include multifaceted response approaches to enable survivors or people and communities at risk to avoid GBV and the negative effects it has on individuals, families, and communities. These responses include psycho-social counseling and psychotherapy for rape and sexual assault survivors, support groups, and social work support (e.g., *Rape Crisis*’ rape counseling services), where individuals’ psychological health and the abilities to cope are targeted by professionals who are equipped and trained to deal with traumatic events such as rape and assault. Shelter services aim to help women and children leave unsafe spaces where violence can occur and provide them with safe living spaces, resources, and skills to empower themselves (NSMSA, 2020). For example, while providing shelter for survivors, the Mpumalanga Shelter Service also implements economic empowerment and skills development programs to ensure that women are no longer dependent on perpetrators (Booysen, 2019). Skills training is a widely implemented response strategy. It includes economic (e.g., banking and budgeting workshops), agricultural (e.g., subsistence farming skills), psycho-social (e.g., strategies for coping), and professional skills (e.g., skills that can render income such as typing, driving, and beauty therapy). Other secondary and tertiary prevention interventions employed in South Africa include medical and legal aid services and advocacy work.

### The Funding of GBV Interventions

Most NGOs in the Global South depend on donor agencies and funders for their survival (Elbers & Arts, 2011). To qualify for funding, NGOs typically must meet various funding conditions. Dworkin and Barker (2019) highlight that it is critical to consider how the constraints and conditions of funders influence intervention design and cause some NGOs to change their interventions radically. Through their funding conditions, donors are known to exercise influence over NGO project design and implementation and set requirements for monitoring and evaluation (M&E), reporting, and allocating funding to specific activities (Ismail, 2019).

Elbers and Arts (2011) explored the potentially adverse effects of donor conditions on NGOs and the strategies they employ to deal with GBV. They found that donors prescribe the types of projects they are willing to support, the target groups, the strategies, and the geographical areas. Dworkin and Barker (2019) argue that program content is often altered by donors who stipulate the types of activities and overall program designs they are prepared to fund. With NGOs compelled to pursue funding, there is evidence that some voluntarily adapt their program activities accordingly and even become involved in areas where they have little experience (AbouAssi, 2013).

The second aspect affected by funder requirements, outlined by Elbert and Arts (2011), relates to accountability. Most donors expect periodic financial and narrative reports on the progress and performance of projects towards precontracted goals and intended results against indicators, timelines, and budgets (Elbers & Arts, 2011). These accountability requirements and power relationships thus affect the M&E practices of South African NGOs (Agyemang et al., 2017; see also Mueller-Hirth, 2012).

Finally, funders often earmark their funds for particular expenses and do not allow the organizations to use funds for overhead costs (e.g., human resources and fundraising), which policy stance ultimately impacts NGOs’ sustainability (Cammack, 2013).

However, it is important to acknowledge that funding relationships exist on a continuum, with NGOs responding to funder demands in diverse ways. Responses range from full compliance to negotiating mutually agreeable terms or, in some cases, severing ties altogether (AbouAssi, 2013). Some NGOs also engage in forms of “micro-resistance” (Richards, 2008), such as submitting selective or inaccurate reports, fabricating documentation, or using humor (cynical remarks and satirical laughter) to subtly challenge funder demands while maintaining outward compliance (Girei, 2023). These dynamics highlight the complexity of funding relationships, demonstrating that NGOs are not merely passive recipients of donor influence but actively navigate and, at times, resist imposed constraints.

### Research and Collaboration

Throughout the literature, there are calls for GBV programs to be evidence-based, that is, built on what has been found to be effective, informed by theoretical models, guided by formative and pilot research, and multifaceted to address several causal factors (SaferSpaces, 2020). This necessitates the learning of organizations through the use of external research.

Collaborative studies between NGOs and external researchers highlight the need for sufficient prior agreement on the mutual expectations regarding the contributions of the study and research principles (Olivier et al., 2016), together with continuous feedback and reflection involving the host organization, research team, and end-users of the interventions (Farnworth & Vaarst, 2012). Successful and sustainable NGO-researcher partnerships are characterized by trust, transparency, respect, and mutual contributions (Rathgeber, 2009). The key challenges in this relationship are asymmetrical power relations, lack of recognition and respect for the contributions of each partner (Olivier et al., 2016), and divergent goals and approaches (Rathgeber, 2009).

Anecdotal evidence suggests that NGOs in the GBV sector are not only failing to collaborate with researchers but also with other service providers in their domain. Informal discussions with stakeholders in the South African NGO sector indicate that many organizations operate in isolation, with limited engagement in collaborative research or shared learning initiatives (Anonymous, personal communication, April 15, 2021). Staff members from GBV NGOs have highlighted a lack of structured collaboration, infrequent sharing of evaluation outcomes, and an absence of best practice frameworks that could strengthen collective impact. Additionally, there appears to be a disconnect between academic research and practical implementation, with NGOs often lacking the resources or networks to engage meaningfully with external researchers. This fragmentation within the sector limits the potential for evidence-based interventions and the development of a cohesive intervention community.

## Method

### Research Design

This study employed an exploratory qualitative research design. Data were sourced from nine semi-structured interviews with GBV NGO staff across South Africa, to obtain detailed accounts of the inner workings of NGOs and the contextual drivers of their decision-making.

### Sampling Strategy

Given the limited research on this topic within the GBV sector in South Africa, the literature indicated that an initial investigation should focus on NGOs and their perceptions of key drivers and barriers, rather than on funders’ motivations and practices. Existing research suggests that funder-NGO relationships can be volatile. To fully explore these dynamics, it is essential to examine each stakeholder separately. Consequently, this study was designed to interview NGO staff members as a first step. The selection criteria for our participants were individuals working in a funded South African GBV NGO (preferably in M&E or program management).

We identified 25 possible GBV NGOs in South Africa as potential participants for this research. Initially, we reached out to a contact in our network who worked at a GBV NGO, and they connected us with our first interviewee. From there, we asked each interviewee to suggest additional individuals we could contact within their network. Sometimes, interviewees shared contact details directly or facilitated introductions via email. Using this non-probability snowball sampling approach, we contacted 32 individuals, of whom nine from different GBV NGOs agreed to be interviewed.

### Sample

Table 1 indicates the location of the participants’ NGO and the types of GBV interventions provided. Five of South Africa's nine provinces were represented in the sample.

**Table 1.** Data Providers.

Province	Number of NGOs	Types of GBV Interventions Provided
Western Cape	3	Legal, psycho-social support, and training Shelter Training and advocacy
Eastern Cape	1	Legal assistance and shelter
Gauteng	3	Counseling, training, and shelter Medical and legal services Psycho-social, legal, and shelter support services
Mpumalanga	1	Shelter and counseling
KwaZulu-Natal	1	Counseling and training

### Data Collection and Analysis

The nine semi-structured interviews (eight via Zoom and one in-person) were conducted to gain comprehensive insights into the operations of GBV NGOs. The interviews’ duration ranged from 45 min to 1 hr, and no incentives were provided for participation. Thematic analysis was used to analyze the transcribed interviews.

The development of the interview questions was informed by key influencers of decision-making that emerged from the literature, as well as gaps identified by the researchers (trends and practices). The literature review revealed a lack of published and publicly available evaluations of GBV NGO interventions in South Africa. As a result, interview questions regarding organizational evaluations were necessary to determine whether evaluations are infrequent or whether organizations conduct them but do not publish or share the findings. Additionally, while anecdotal evidence suggests limited collaboration among organizations, the literature primarily discusses the consequences of competition rather than the underlying reasons for this lack of collaboration. Therefore, the interview questions aimed to explore the factors contributing to limited collaboration and its impact on the GBV sector. Lastly, the interview questions focused on the NGO-funder relationship as well as general challenges of operating in this sector.

To analyze the data qualitatively, Braun and Clarke's (2006) steps for realist thematic analysis were applied. This study adopted a theoretical thematic analysis approach, enabling the researchers to analyze data based on predefined research prompts. A deductive thematic analysis method was employed, as it allows for a more detailed examination of specific aspects of the data, with themes guided by the researchers’ theoretical and analytical interests (Braun & Clarke, 2006). Thematic analysis was conducted at the latent level, extending beyond the semantic content to identify underlying ideas, assumptions, and conceptual frameworks that shape the data (Braun & Clarke, 2013).

### Positionality

This study was inspired by the ongoing issue of GBV in South Africa, particularly in the wake of Uyinene Mrwetyana's murder—a 19-year-old student raped and killed by a post office worker in August 2019 (Mogoatlhe, 2019). Her death ignited the #AmINext movement, and nationwide protests demanding urgent government action against GBV (Johannes, 2019). During this period, we personally experienced the grief, anger, and activism that followed, making the issue feel deeply immediate.

Although we had no personal experience with GBV and no prior working relationships with NGOs in this sector, these events heightened our awareness of the critical need for GBV prevention and intervention efforts. This motivated us to explore the role of NGOs in addressing GBV, with the aim of contributing meaningful insights to the field. Acknowledging our position as outsiders to NGO work, we remained reflexive throughout the study, critically engaging with the data while ensuring sensitivity to the experiences of those working in the sector. We made a conscious effort to maintain a neutral perspective, allowing NGO professionals to share their lived experiences without undue influence. This was particularly important during the development of the interview guide, where we avoided leading or value-laden questions, and during the interviews themselves, which followed an open-ended, participant-led format that enabled professionals to prioritize what they felt was most significant.

### Ethical Approval and Considerations

This study received ethical approval from the Commerce Faculty's Ethics in Research Committee at the University of Cape Town (Ref: REC 2021/03/016). Participation was voluntary, and participants were assured of anonymity. Participants were assigned a number, and any identifying information was removed when reporting the findings.

## Findings

The thematic analysis revealed four themes: (1) a focus on secondary and tertiary initiatives, (2) the loss of organizational autonomy, (3) overburdened staff, specifically regarding M&E practices and reporting, and (4) difficulties in fostering collaboration and organizational learning.

### Theme 1: A Focus on Secondary and Tertiary Initiatives

All nine NGOs in this research offered secondary and tertiary GBV strategies, but none implemented primary prevention strategies. The variety of interventions and aid offered included psycho-social counseling and therapy, support groups for social workers, medical assistance, legal advice, advocacy, workshops and training, and shelter for victims. Interviewees explained that these interventions were aimed at empowering women to remove themselves from adverse situations or to help them recover from acts of GBV and to mitigate its detrimental long-term consequences. While the interviewees expressed a desire to focus more on primary prevention, they indicated insufficient resources to provide interventions on all three levels. Interviewee 2 explained:

When a woman comes through the gates, her perpetrator just moves on to the next victim. So, we're not really getting to the cause of what is causing [GBV], which is men and attitudes of patriarchy. And that's where the community-based interventions are crucial in actually getting to what is underpinning this in the South African society. It's mostly patriarchy and, obviously, a very brutal past that has normalised violence … We should be looking at other prevention areas. One of the things that frustrates me the most is that there still isn't an understanding that we really have to start talking to perpetrators… And I don't believe that we'll be able to start tackling gender-based violence until we start getting girls and boys involved. These are things that we really should be looking at.

While the focus remains on reactive services, several interviewees mentioned the untapped potential of primary prevention. Interviewee 8 remarked that there is a noticeable community interest in primary prevention initiatives targeting men and boys, yet there is limited implementation of such programs:

As for the men and boys’ work … there is interest, even coming from our communities. But we don't seem to be trying to design anything focused on them. I think it comes from … that legacy of … making sure that women are taken care of and empowered [and] elevated. I just think it would be too difficult for people to change that now.

Similarly, Interviewee 4 described the current service model as predominantly reactive:

[It is] like … an afterthought … such that the service that we see out there … is like the reaction to what is happening … You hear the number of women who are murdered … then the services provided [are] reactions to what has happened … but there's no investment in how socialization of males in the country is done. So unless and until [we invest in] the socialisation of boys in behaving as men … we're going to continue reacting to it … So my take is we are reacting rather than investing in what could be the solution to the problem in this country.

Interviewee 5 further emphasized that, although there are some engagements with men and children, the psychosocial needs of women and girls are prioritized, leaving limited opportunity to challenge patriarchal norms:

I'm not saying we don't work with men and children. However, the way in which we work with them doesn't prioritize them and their psychosocial needs [but] prioritizes the psychosocial needs of women and girls. [The] way in which we work is to ensure that … there's education and understanding and … dialogue … but not so much in terms of ensuring that we look at … working to change patriarchal attitudes of men. [It's] important and the work needs to be done [but] that's not something that our organization engages in anymore.

In developing countries like South Africa, resources to address violence are scarce. Consequently, many women seek assistance from civil society following events of GBV, specifically the services offered by NGOs, which may be why secondary and tertiary GBV interventions are the predominant responses. However, it is concerning that the data from this study suggests that there are limited efforts by the NGOs interviewed to create a healthy foundation of societal change to lessen the need for secondary and tertiary support.

These findings also illustrate the inherent tension between addressing the immediate needs of GBV survivors through secondary and tertiary interventions and the need for primary prevention strategies that challenge the underlying causes of GBV. Despite the evident community demand for a broader, proactive approach, the NGOs in this study continue to emphasize reactive interventions—a focus rooted in both limited resources and a longstanding commitment to feminist principles.

### Theme 2: Loss of NGO Autonomy

A prevailing theme across all the interviews centered on the loss of autonomy experienced by NGOs. This pertained to the diminishing power and freedom of NGOs to make decisions regarding program design and implementation, the allocation of funds, and the duration of programs. Interviewees frequently expressed sentiments such as “losing our autonomy,” “they own my program,” and “we’ve lost our sense of ownership,” highlighting the power dynamics inherent in NGOs’ relationships with funders.

This loss of autonomy was primarily attributed to the relationship between the funder and the organization, particularly the power of funders to impose strict requirements on NGOs. This theme had two sub-themes: *Dictating finance use* and *dictating program design and implementation*. These sub-themes underscore the unequal power dynamics between funders and NGOs and their consequential impact on the operations and potential effectiveness of NGOs and their interventions.

#### Dictating Finance Use

Five interviewees explained that their NGO experienced a loss of autonomy due to restrictions imposed on the utilization of funds, which led to complexities and deficits. Some funders specified what funds could not be allocated to, such as electricity or administrative and operational costs, while others did not allocate enough for particular areas, such as salaries. Interviewee 3 stated:

…[we need] general funding … I mean, you need money to keep lights on, you need money to buy food and fuel, and that type of thing, which you don't necessarily get if [funders] are specific in what they are funding … [Funders] should say, “Let's rather let the actual people that have to work with us on a daily basis decide where the funding should be used.”

Interviewee 5, from an NGO, focused on upskilling victims of GBV to ensure their economic freedom from their perpetrators, revealed that the NGO's funds were restricted to training only one representative coordinator to provide technical assistance to women embarking on self-sustaining entrepreneurial activities. The trained coordinator was then tasked with training approximately 20 others, which resulted in the “heavy dilution” of skills.

#### Dictating Design and Implementation

Three interviewees described program design as non-negotiable and dictated to NGOs, which also influenced the implementation of their GBV programs. Participants elaborated on how these prescribed requirements affected various processes within NGO operations and diminished their autonomy to apply the expertise and skills they have acquired through experience in and familiarity with the South African GBV context.

The interviewees highlighted that, despite their skills and expertise, funders assumed a dominant role, dictating actions to an extent that undermined NGOs’ autonomy. Interviewee 7 remarked:

…because they bring in the money, you seem to lose the autonomy, despite the fact that you bring in all the experience, expertise, and skills because they are now stipulating how many people you employ, what your programme should do, how it should be done, and, unfortunately for us, where it should be implemented.

This interviewee provided the example of how an area in Gauteng, with an exceptionally high incidence of GBV but minimal cases brought before the courts, was excluded from the scope of the NGO's legal assistance intervention due to the funder's directive to focus on rural areas. Interviewee 6 held the same sentiment: “The moment they give you money, you find that they become prescriptive and basically have a sense of ownership over how you steer the ship…”

Three interviewees also highlighted that design or implementation changes were often made to ongoing programs to secure funding for future activities. Funders may have specific objectives that closely align with those of the NGO, but adjustments to the design or including additional elements are necessary to meet the funder's requirements. Interviewee 5 provided an example, explaining that a counseling service that had been running successfully for years needed to incorporate a new element to secure funding from a new funder. Specifically, the funder required that the NGO include an HIV-related intervention alongside the existing rape counseling services, aimed at leveraging the NGO's reach and resources to implement a health intervention alongside a GBV intervention. The NGO experienced a loss of autonomy, as it had to alter its program design to incorporate the agenda of the funder.

At times, NGOs find themselves compelled to modify their programs because funders have changed the fund allocations or objectives without consultations that would allow negotiations, input, and suggestions or the balancing of priorities, leaving the NGO staff feeling undermined, which impacted their sense of ownership of the programs. Interviewee 3 explained:

…we said that we would like to use [the funds] as part of our skills development programme, and then they came back after [awarding us the funds] and said, “No, but it has to be environmentally friendly, and it has to be rural women. … We are currently revising our whole project … Because [the funder] said ‘environmentally friendly’, we have to include something like vegetable gardens … All this change needs to be done quickly while still implementing the urban programmes as before.”

The scenario described above illustrates how an NGO was compelled to expand its operations to rural areas, retrain its skills development trainers, and overhaul its implementation strategy to adhere to new funding requirements. Interviewees also noted that funders are aware that NGOs in South Africa, particularly those in the GBV arena, often have no choice but to accept and implement changes imposed by funding requirements due to the urgent need for funds.

Moreover, all nine interviewees emphasized a common practice of designing interventions with the funders’ specific requirements in mind, allowing the funders to dictate decision-making within the NGO to a large extent, thereby subjugating the agency of NGO staff. The insufficient funding allocated to essential areas such as daily operational costs, ongoing technical and training support, and market-related salaries adds to the already significant pressure on NGOs, which, in turn, fosters resentment towards the funders.

Donors are known to exert influence over NGOs’ project design and implementation, particularly by earmarking funding for specific activities (Elbers & Arts, 2011). Nearly all donors stipulate requirements outlining the type of projects they aim to support, including themes, target groups, strategies, and geographical areas. To secure funding, it is not uncommon for NGOs to align with donors’ funding conditions and adapt to their intervention strategies, irrespective of what they deem necessary to address issues (Dworkin & Barker, 2019; see also AbouAssi, 2013).

### Theme 3: Overburdened Staff: Reporting and Evaluation Challenges

All interviewees underscored the strain placed on NGOs by the overwhelming workload and insufficient support and funds to meet these demands. Data obtained from four interviewees indicated that funders’ excessive reporting requirements often result in unnecessary strain on the NGO. Interviewees noted that funders typically demand monthly or quarterly reports and sometimes require NGOs to submit yearly proposals even after having secured multi-year contracts. While interviewees deemed yearly reports necessary to “keep the funder in the know about where their funds have been used” (Interviewee 8), Interviewee 6 described the reporting intervals as “completely excessive,” with some requiring a report every month. Interviewee 9 explained:

…[the new reporting] created so much extra work for the counsellors, for the people who have to capture that, and then for [the M&E specialist], who has to put all of that together for [the funder] … It's a ton of work … and then the funders also don’t pay overheads, so if it takes you three hours extra to do data capturing, it's because it's not part of our existing structures or systems, but we won’t get paid for the shortfall.

This excessive reporting has the detrimental knock-on effect of diverting NGOs’ attention from their core activities. Interviewee 1 highlighted how stringent reporting requirements imposed by funders hindered the NGO's impact by consuming valuable time and human resources that could be better spent on improving and implementing interventions for survivors of GBV.

Interviewees remarked that funders often impose stringent requirements for evaluation using indicators of success. Two interviewees highlighted that international funders often prioritize numerical targets, which the interviewees attributed to a lack of understanding of and sensitivity to the local context. Interviewee 7 explained:

With the international funders, … all they are looking at is the ending sites [numbers]. They want targets, and they want to report to their counterparts in the US that … they’ve reached these numbers… I found that, in the US, there isn’t that level of understanding, empathy, or support. We find it quite challenging working with international funders.

Four interviewees noted that discrepancies between the indicators of success used by funders and the NGO posed challenges and suggested that NGOs are forced to either change their indicators or collect data on indicators that were not initially incorporated in the planning. This divergence in indicators was due to funders requiring specific metrics to verify impact in line with their objectives or goals. Interviewee 5 remarked: “…we have new indicators that are entirely unrelated to our program plan.”

Interviewee 4 articulated the complexity and challenge of balancing the NGO's original GBV indicators with the need to accommodate the funders’ indicators, which requires time-consuming collection, analysis, and reporting of data. The human resources required lead to diluted efforts in other areas, including the collection and monitoring of the data the NGO requires. Interviewee 4 explained:

…[the funder] won't count the fact that I've given [the survivor] support. They will count if she agrees to get tested [for HIV], and she takes PrEP and all of that, you know. My normal work, which is providing counselling and ensuring the well-being of the survivor, doesn't count. … So, I have to incorporate what they want in order for me to get access to the money. This is a big challenge because now I have to manage my monitoring and evaluation officer because I must remind them, “The donor wants it, don't forget.”

Five interviewees also noted a significant challenge in the form of a shortage of funds and resources to conduct evaluations to demonstrate impacts. While most interviewees acknowledged that evaluations remained a necessary practice, they noted that there was either insufficient funding to cover such evaluations or the allocated funds were inadequate to conduct large-scale, independent longitudinal evaluative studies. Interviewee 5 said:

…donors want to see these high impacts, but they don’t pay for the longitudinal study… They have great indicators that they want for evaluation. They want to know quality, qualitative feedback, and proof of impact. But they don’t pay us to carry out the evaluation exercises. And they forget that we need people and time to run interviews and to do survey questionnaires… Essentially, all those things need time and human resources… But they don’t give the money towards [M&E].

Interestingly, and linked to both Themes 1 and 2, the funders’ choice of indicators of success influences the type of intervention implemented, its design, and its implementation. Interviewee 5 explained:

It's easy for the secondary response work to be funded because we can count the victims, so we're going to just push all our money to secondary [prevention]. But we actually really need primary prevention… Funding is about egos, … and “My ego is not going to be great because you can't show me that you've changed 50 000 people's attitudes, but you can show me 50 000 survivors who you've helped, you've counselled. If you can quantify it, I'll give you the money.”

Despite these efforts, the interview data show a notable lack of impact evaluations. This can be attributed to various factors, such as limited human and financial resources, a lack of will, and time constraints. The focus seems to be on the utilization of monitoring data to provide current information on program operations aimed at enabling swift and impactful changes.

Agyemang et al. (2017) and Mueller-Hirth (2012) indicate that the stringent short-term accountability measures imposed by funders undermine the potential impacts of NGOs. The measurement of the indicators required by funders demands capacities that are outside the core expertise of NGOs, adding pressure to an already under-resourced sector (Mueller-Hirth, 2012), and funders fail to allocate funds necessary to conduct large-scale and informative evaluations. This leaves NGOs with a lack of information to ensure ongoing agility in their responses and improvements to their programs (Mueller-Hirth, 2012). All these factors coalesce in tensions between NGOs and their funders (Agyemang et al., 2017; see also Elbers & Arts, 2011).

### Theme 4: Struggles of Collaborative and Organizational Learning

This theme addresses the challenges identified regarding collaborative and organizational learning in the GBV sphere. While the interviewees recalled instances of successful collaboration and knowledge-sharing, eight of the nine interviewees highlighted areas where collaboration could be improved and provided examples of factors hindering successful collaboration. Collaborative learning encompasses ongoing joint efforts and information-sharing among role players through the communication of proven best practices and lessons learned. Interviewees noted that learning and collaboration could be greatly enhanced by independent research and evaluations utilizing informal needs assessments and internal M&E results, which should be applied in organizational decision-making. This theme comprised two sub-themes: *Working in silos* and *Prioritizing internal learning: Valuing experience over external knowledge*.

#### Working in Silos

This sub-theme highlights the problem of South African GBV NGOs restricting their communication and collaboration to those within their own organization or department. The data revealed the disadvantages NGOs face by not openly communicating and collaborating with each other, along with the underlying reasons for this culture. These reasons include a scarcity of resources and technical expertise, funders’ reluctance to fund collaborative efforts, and competition among role players in the sector.

Six interviewees indicated that the lack of information-sharing between NGOs through online resources, informal channels, or formal pathways is primarily due to it not being a priority. The foremost concerns revolve around meeting intervention targets and securing funds for various interventions, overshadowing the importance of disseminating information. Interviewee 9 said: “There's no active sharing because it falls off the radar because they’re so busy doing other things.”

Interviewee 2 agreed:

From our side, it's an issue around just the time and the ability to share those kinds of documents more widely. We would definitely like to share it more widely because … if our documents are valuable to the next organisation, and it's able to help women from a different geographical location, then that's awesome. But, at the end of the day, … our first priority is our programmes, not other people's programmes.

Four interviewees added that their organizations refrained from engaging in regular online or formal publishing due to resource constraints. They noted that they lacked the human capital and technical expertise or the financial means to hire personnel to manage their websites and disseminate information. Interviewee 9 explained: “…[our] knowledge-sharing is awful. I mean, we've had three national roundtables; reports have been written for all of them, but none of them have been shared. I don’t think we even know who to contact about that, who can help us get it out there online.”

Moreover, interviewees highlighted the role of funders in perpetuating the issue of NGOs working in silos. Five interviewees emphasized that collaborative efforts are only pursued if explicitly mandated by funders. Interviewee 4 stated: “We are very donor-driven, so if a donor doesn’t require [collaboration], we don't do it.” Similarly, Interviewee 6 said: “I think we all know we work in silos. It's because we are donor-driven, and it's difficult to collaborate … often, donors, as much as they talk about collaboration, they do pit against each other … If they’d fund collaboration, we are ready to do it.”

Competitive dynamics in the sector was another issue raised as a reason for not sharing information and engaging in collaboration. Five interviewees noted that, due to limited funding, NGOs are hesitant to share what makes them unique, successful, and attractive to funders for fear of losing funding to competitors. Interviewee 1 said:

You won’t find best practices [because] you’re dealing with a sector where you’re so vastly under-resourced and there is so much competition for funds… It boils down to this: “This is my model, and I have to hold on to this model because that is what distinguishes me from every other NGO in the Western Cape. So, if I put this up on my website, online, I speak too much about it, somebody else is going to take it over, and before you know it, I’m going to start losing funding.”

Interviewees expressed a desire to collaborate in addressing GBV or at least acknowledged the importance of collaboration. However, they indicated that there is a link between working in silos, insufficient resources for sharing, and competition, with each aspect reinforcing and perpetuating the others. In a sector where organizations are “fighting for funding” (Interviewee 9), the deliberate practice of organizations “holding things close to [their] chest” (Interviewee 1) cultivates a culture that is “dog-eat-dog, competitive, and weirdly secretive” (Interviewee 9). When asked about potential facilitators of change regarding information-sharing and competition in the sector, most interviewees suggested that funders could encourage and finance collective efforts or ensure the availability of more funds. However, they acknowledged this was not likely.

In support of the need for collaboration, Interviewee 5 praised a funder for actively promoting information-sharing and networking within the sector:

The thing I could give [funder] is that they do create those [information sharing] platforms, or they're trying to create those platforms. They have quarterly meetings where they bring all their “fundies” together. Naturally, you're going to have crossover and discussion and all of that.… you meet other organisations doing similar things, as well as struggling with the same project. And then you do share and learn from each other.

Despite the challenges outlined earlier, certain forms of informal and formal collaborative and organizational learning do occur within the sector, with interviewees noting that these yield positive outcomes for all stakeholders. Informal methods encompass informal interactions, such as staff members from different NGOs engaging in conversations and seeking advice from one another. Interviewees noted that, in these encounters, information is readily shared when requested and that staff members from various NGOs often communicate informally to exchange insights and guidance. Notably, two interviewees emphasized the role of friendship or chance encounters in fostering information-sharing between organizations.

Six participants detailed formal sharing strategies that their NGOs employed or common collaborative methods in the sector that have proven beneficial in the past and recommended that these be more widely adopted. These formal information-sharing practices involved sharing reports, best-practice recommendations, organizational insights, and sector-specific information or research. Interviewee 7 outlined deliberate efforts such as holding YouTube live events and webinars to share information to enhance practice and motivate donor engagement.

Cooper and Shumate (2012) found that when NGOs work collaboratively, they can enhance the quality of care for GBV clients by referring them to NGOs with different specializations. The present study found that, despite recognizing the benefits of partnerships, the competition within the network (competing for volunteers, staff, and funding), funder influence, and a lack of organizational resources and capacity are notable constraints that prevent this much-needed collaboration. Atouba and Shumate (2010) highlight that these issues pose significant barriers, particularly for NGOs in the Global South, which often have less financial and social capital than their Northern counterparts. Shared funding sources could enhance connections between NGOs. Atouba and Shumate (2010) note that NGOs with similar funding sources are more inclined to network as they have comparable statuses or reputations, which mitigates their viewing of other NGOs as a threat.

#### Prioritizing Internal Learning: Valuing Experience Over External Knowledge

This sub-theme encapsulates how participants described their methods of and challenges to organizational learning. They conveyed a sense of internal learning being prioritized over external learning (e.g., academic research), framing it as “us” (internal learning) versus “them” (external learning) when receiving and acting upon feedback. Learning from within their NGO was perceived as more valuable than learning from external sources. For example:

But using the findings [of formal evaluations] is a bit difficult. Because the external evaluations, … they expose systemic issues and difficulties, and maybe which processes could be better, and so on. So, that is fed back to the staff, but it's not acted upon as quickly, and is not nearly as effective as informal internal reviews, which are more collaborative, and a discussion. (Interviewee 5)

Interviewees alluded to effective organizational learning requiring feedback mechanisms to communicate M&E results, including reflections on experiences and context, as well as input from staff during meetings. Four interviewees highlighted that these strategies had been instrumental in enhancing organizational decision-making regarding program design and implementation:

We evaluate what we've monitored. So, we've got a theory of change with the most significant change, pre- and post, and we do qualitative and quantitative research or at least monitoring. But we also have a very strong quantitative process around the most significant change, theory of change, and appreciative inquiry. We make decisions informed by these data. (Interviewee 4)

So, all of our programmes do have a monitoring-and-evaluation component built into it, and that's actually how we learn, and we are able to modify or change or design programmes based on what is coming out of our monitoring-and-evaluation initiatives…. (Interviewee 2)

Three interviewees revealed that observations are a highly valued form of informal organizational learning. These NGOs adapted their design or implementation based on observations of their clients and the community. This involved addressing the needs of target populations directly and staying attuned to their concerns. Interviewee 6 described this as “just keeping an ear to the ground.”

If I say that one amazing thing that really would inform [NGO]'s programme and direction … [it] would be: *What are the requirements of the survivor on the ground?* The women that are coming through the door, what are their needs, what is that telling us about how we can either curtail, restrict, embroider, extend, or put a completely new programme into place? [This] is information that we can recognise faster than researchers. (Interviewee 1)

Internal learning strategies received high praise from interviewees. Still, it was evident that external learning did not evoke the same level of trust and positivity, with five interviewees expressing negative views of external sources of organizational learning. Interviewee 7 remarked: “Researchers don't get it and only report what makes them look good.”

Interviewee 4 stated:

[Research] is mostly geared for the North anyway. What's happening is [the researchers] sometimes think they know best, but they have academized it.… and I blame researchers because then they do this fancy research published things that are far removed from the reality of the people that actually need interventions. And then the funders look at this research and prescribe all sorts of nonsense. … I’d rather we just do it in-house.

The limited use of academic and scientific research to guide best practices was unexpected, considering the sector's complexity and the importance of NGOs’ engagement with independent research (Ormel et al., 2020). Olivier et al.'s (2016) findings resonate with the sentiments expressed in this study: NGOs distrust and are reluctant to implement recommendations based on academic research. NGOs also tend to devalue external research when they feel that researchers have not adequately involved them or considered the sector's context and realities (Farnworth & Vaarst, 2012). This distrust may lead to outright rejection without proper consideration of what may be valuable research contributions (Ormel et al., 2020). Academic and external researchers typically prioritize knowledge expansion and dissemination through scholarly publications driven by academic promotion expectations, norms, and priorities (Rathgeber, 2009). NGOs, on the other hand, seem to prioritize the practical utility rather than the theoretical underpinnings of research (Olivier et al., 2016). The out-of-hand dismissal of academic research was evident in Interviewee 4's characterization of research as “fancy research published things.” The “us vs. them” perspective underscores NGOs’ inclination to prioritize internal learning, drawing from their own staff and data.

## Discussion

**Figure 1.**
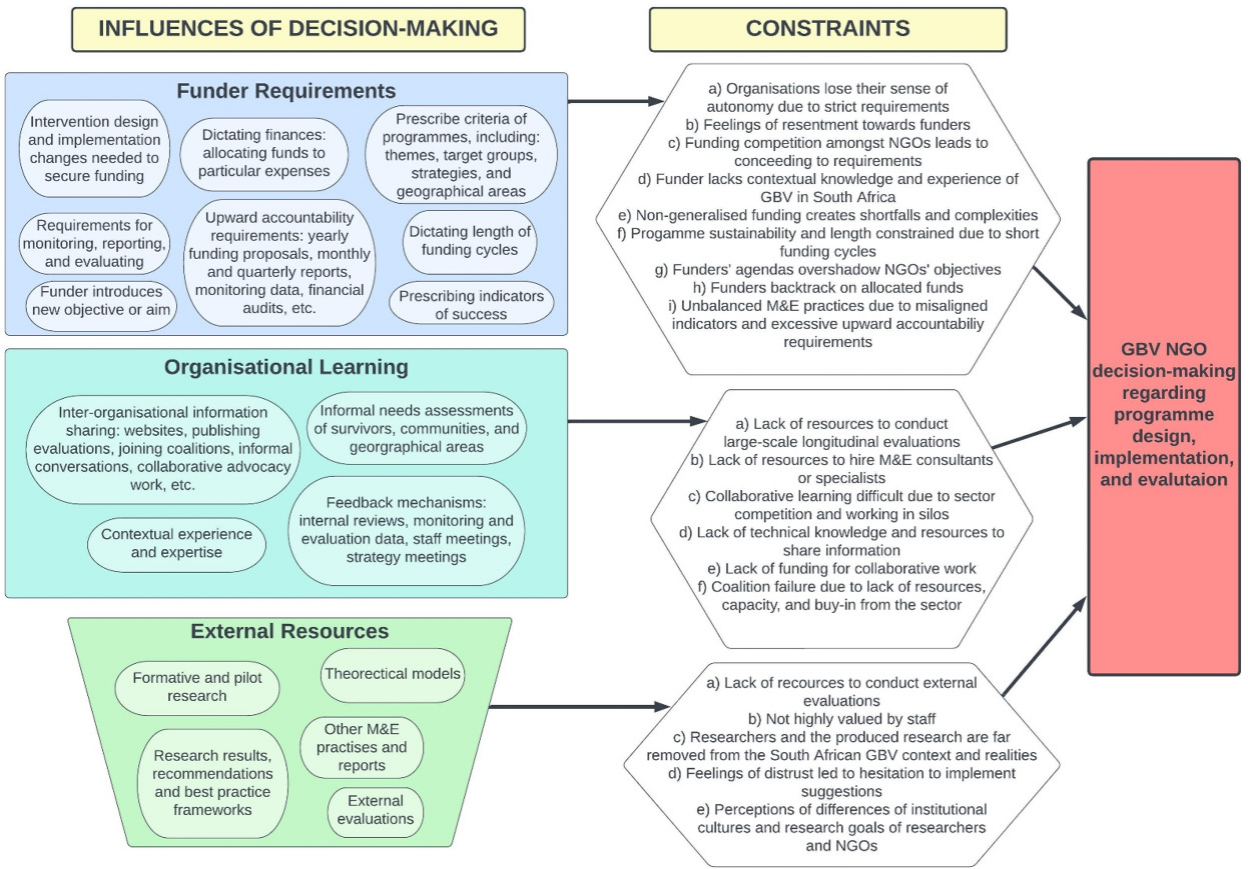
Factors Influencing the Decision-Making of GBV NGOs in South Africa.

*Source*. Kieser (2023).

The findings of this study indicate that the trends, practices, and drivers of decision-making in the GBV NGO sector are shaped by funder requirements and organizational learning through practice and reports, with little attention to other information sources. Each of these, however, poses complexities and challenges for NGOs. Figure 1 summarizes how decisions are influenced and the constraints that emerge from the data.

The data revealed that funder requirements and internal organizational learning are the key influencers of trends, practices, and decision-making and are the main forces configuring the South African GBV sector. While NGOs do rely on external resources for information gathered through informal interactions, there is a pervasive distrust of academic research findings. Recommendations for practice are made based on these findings.

### Suggestions for Practice

It is clear that funder demands greatly impact NGOs’ autonomy. While funders are entitled to determine the nature and execution of programs, greater collaboration between the parties is required to ensure that initiatives address real needs and that NGOs retain a sense of autonomy and, thus, their agency and commitment. Collaboration between NGOs and funders will reduce tensions in this relationship, prevent resentment towards funders, and enhance NGOs’ capabilities through optimized operations, with a positive impact on the relevance and impact of programs.

In addition, challenges related to the allocation of funds were identified. Core funding provides NGOs with general financial support to carry out their activities and functions, whereas project funding is allocated for specific initiatives and cannot be used for other purposes. Unlike core funding, project funding typically excludes administrative and basic organizational costs. Project funding often gives more control to the funder, while core funding grants the organization greater autonomy, flexibility, and, ultimately, power. Project funding is usually short-term and secured for a limited period, whereas core funding tends to be long-term (Gibson et al., 2007). The lack of secured, flexible funding often leaves organizations underfunded, contributing to worker stress and high staff turnover, as employees may face irregular or incomplete salary payments (Gibson et al., 2007). Additionally, project funding fosters a competitive environment among NGOs within the same sector, as they vie for limited resources instead of collaborating (Gibson et al., 2007).

This dynamic was evident in our study, where interviewees highlighted the challenge of securing core funding despite needing financial support for essential operational costs such as administrative expenses to keep their organizations running. Instead, they primarily received project-specific funding, which, while valuable, restricted their ability to allocate resources flexibly. Additionally, interviewees echoed concerns about the competitive nature of funding, noting that NGOs often compete for limited grants rather than working collaboratively to address GBV more effectively.

Funders also need to be aware of the financial and human resource costs NGOs incur in collecting the required data and reporting on outcomes. This needs to be addressed with a view to preventing resource-constrained NGOs from expending excessive amounts of time and money in meeting funders’ requirements, leading to a loss of focus on the intended positive impacts of the programs.

Funders could adopt greater flexibility regarding the allocation of pledged funds in consultation with NGO staff who have practical experience in the operations of NGOs. Collaboration in M&E and impact studies will ensure that the outputs are helpful to both parties and are applied in the ongoing refinement of initiatives.

The findings further show that greater collaboration and knowledge-sharing among groups of funders and NGOs are required to ensure that programs are designed around the real needs of the community. This requires a holistic approach to prevent both overlaps and gaps in programs in practice. Funders need to be informed of the need for programs that are vital to ensure comprehensive addressing of GBV, such as prevention initiatives. While the outcomes of prevention initiatives are difficult to quantify, not addressing the fundamental causes of GBV leads to a gaping hole in what should be a concerted effort. While there are some primary interventions and awareness and advocacy campaigns in South Africa to foster non-violent attitudes and behavior among men (e.g., MenEngage, One Man Can, MenCare+), the sampled NGOs’ GBV efforts were focused only on secondary and tertiary interventions because funders require reporting on specific indicators. This seems to have led to the picking of low-hanging, highly visible fruit. No amount of secondary and tertiary interventions could ever completely undo the trauma of victims of GBV, and neglecting prevention not only amounts to attention after the fact but also increases the need for secondary and tertiary services, which are already overburdened in South Africa.

The imposition of upward accountability requirements by funders was observed to create internal imbalances within organizations, escalate pressure through heavy workloads, and influence decision-making about evaluations. The allocation of time and resources towards donor-led monitoring detracts from NGOs achieving their primary objectives and their ability to evaluate their work. This indicates a need to foster communication and negotiation between NGOs and funders regarding accountability measures.

The findings indicate that funders must gain a deeper understanding of the contextual nuances surrounding program design, intervention implementation, and NGOs’ operations. Specifically concerning international funders, it is evident that GBV staff often perceive that their expertise in and first-hand experience of the South African context are disregarded and underestimated. Funders should give greater consideration to the strategies that they embark on, which require consultation with NGO staff on the ground.

A significant finding is that initiatives to prevent and address GBV could benefit significantly from information-sharing among all stakeholders. This would require the allocation of funds specifically for information-sharing strategies, such as disseminating evaluation results and best practices through online platforms and in-person events across the sector. Funders could also directly support coalitions and initiatives that promote collaboration among funders and NGOs.

This study underscores the importance of NGOs preserving their autonomy, program integrity, and organizational effectiveness in harmony with the aims and requirements of funders (see Dworkin & Barker, 2019; see also Ismail, 2019). This requires open and honest dialogue and ongoing collaboration. NGO staff must enjoy a sense of ownership and the necessary control of programs to maintain their dedication to what can be emotionally draining work.

In terms of collaboration and information-sharing, various factors hinder organizations from sharing more online and making information readily accessible to the public. These barriers include a lack of organizational capacity, resources, technical expertise, and concerns about the potential misuse of what they consider intellectual property, which could lead to loss of funding. While collaborative spaces and coalitions have the potential to foster trust and sharing, the recent inactivity of The Shukumisa Coalition (a national network of over 60 South African organizations advocating for effective, survivor-centered responses to sexual violence) exemplifies the challenges identified in this study, including a lack of willingness, capacity, and funding in coalitions in the GBV NGO sector. Organizations should recognize the advantages of information-sharing and collaboration in enhancing the overall impact of GBV NGOs and implement mechanisms to facilitate these practices. A cohesive framework for addressing GBV will go a long way in preventing the overlaps that cause competition for funding and the hoarding of information. The sharing of best practices is crucial in ensuring the long-term success of these programs.

An unexpected finding of this study was the poor perceptions of GBV organizations of external learning through scientific and academic research. Academic publications are not highly regarded in NGOs’ decision-making. Interviewees expressed that researchers are often driven by publishing goals, that focus on theoretical frameworks and academic interests rather than the practical needs of NGOs and the sector. They referred to this as “academicized” issues that prioritize conceptual discussion over actionable insights, making it less accessible or relevant to those working directly in the field. Researchers should note this disconnect between academia and practice and focus on pragmatic research that adds value in the long and short term. They could collaborate more with NGOs and funders to address under-researched practices, and the outcomes of such research should be tied to information-sharing platforms. Such research could provide invaluable input regarding neglected aspects of GBV and program design, as well as solutions to problems encountered in implementation.

### Limitations

This study has several limitations that impact the transferability and interpretation of the findings. Five of South Africa's nine provinces were represented in the sample, as well as nine GBV NGOs. A larger sample size would facilitate more nuanced findings.

Additionally, the sample for this study consisted exclusively of staff members from GBV NGOs. Consequently, the findings are limited to their perspectives. Incorporating interviews with other stakeholders, such as funders, coalition leaders, and beneficiaries, could offer more comprehensive viewpoints.

## Conclusion

GBV affects every person in society, whether directly or indirectly, tearing at the very fabric of society, with a knock-on effect on every level of society and, ultimately, the country's economy. Many of these utterly destructive outcomes are very difficult to measure, especially the psychological outcomes, which include traumatized children. Addressing this phenomenon that significantly impacts societal health and cohesion necessitates a comprehensive approach beyond fragmented, improvised interventions.

Current efforts seem to be focused on only three pillars of South Africa's (2020) National Strategic Plan on GBV, namely (3) justice, safety, and protection; (4) response, care, support, and healing; and (5) economic power. In contrast, there appears to be limited attention to pillars: (1) accountability, coordination, and leadership; (2) prevention and rebuilding social cohesion; and (6) research and information management. This does not bode well for attempts to address the issue holistically and effectively in the long term. If GBV is to be eradicated for the betterment of society, there must be a concerted effort to eliminate competitive dynamics, power struggles, and the embellishment of public relations initiatives.

South African GBV NGOs persist in their endeavors, demonstrating remarkable resilience and dedication in the face of the constraints and challenges illuminated by this research. Their feedback has shed light on shortcomings in the practices prevalent in the sector. By providing insider insights into the operations and challenges faced by these NGOs, we have identified potential opportunities to improve the complex spectrum of NGO functioning, intervention design, implementation, and evaluation. It is hoped that the lived experiences of the interviewees will help to shift the expectations of funders and lead to more coordinated and meaningful initiatives designed and implemented in concert to address GBV.

## References

[bibr1-10778012251348425] AbouAssi K. (2013). Hands in the pockets of mercurial donors: NGO response to shifting funding priorities. Nonprofit and Voluntary Sector Quarterly, 42(3), 584–602. 10.1177/0899764012439629

[bibr2-10778012251348425] Africa Health Organisation. (2021, January 14). Gender based violence fact sheet: South Africa. AHO. https://www.aho.org/news/gender-based-violence-fact-sheet-south-africa/

[bibr3-10778012251348425] Agyemang G. O’Dwyer B. Unerman J. Awumbila M. (2017). Seeking “conversations for accountability”: Mediating the impact of non-governmental organization (NGO) upward accountability processes. Accounting, Auditing, & Accountability, 30(5), 982–1007. 10.1108/AAAJ-02-2015-1969

[bibr4-10778012251348425] Atouba Y. Shumate M. (2010). Interorganizational networking patterns among development organizations. Journal of Communication, 60(2), 293–317. 10.1111/j.1460-2466.2010.01483.x

[bibr5-10778012251348425] Barker G. Ricardo C. Nascimento M. Olukoya A. Santos C. (2010). Questioning gender norms with men to improve health outcomes: Evidence of impact. Global Public Health, 5(5), 539–553. 10.1080/17441690902942464 19513910

[bibr6-10778012251348425] Booysen C. (2019, July 25). NGOs fight for better shelters for women. *IOL News*. https://www.iol.co.za/capetimes/news/ngos-fight-for-better-shelters-for-women-29811581

[bibr7-10778012251348425] Braun V. Clarke V. (2006). Using thematic analysis in psychology. Qualitative Research in Psychology, 3(2), 77–101. 10.1191/1478088706qp063oa

[bibr8-10778012251348425] Braun V. Clarke V. (2013). Successful qualitative research: A practical guide for beginners. Sage.

[bibr9-10778012251348425] Cammack J. (2013). Considered choices for funding decisions: How to calculate the real cost of donor-funded projects; when to say “yes” and when to say “no”. Development in Practice, 23(4), 589–595. 10.1080/09614524.2013.790943

[bibr10-10778012251348425] Carlson J. Casey E. Edleson J. L. Tolman R. M. Walsh T. B. Kimball E. (2015). Strategies to engage men and boys in violence prevention: A global organizational perspective. Violence Against Women, 21(11), 1406–1425. 10.1177/1077801215594888 26202155 PMC4592362

[bibr11-10778012251348425] Casey E. A. Carlson J. Fraguela-Rios C. Kimball E. Neugut T. B. Tolman R. M. Edleson J. L. (2013). Context, challenges, and tensions in global efforts to engage men in the prevention of violence against women: An ecological analysis. Men and Masculinities, 16(2), 228–251. 10.1177/1097184X12472336 25568612 PMC4283930

[bibr12-10778012251348425] Casey E. A. Tolman R. M. Carlson J. Allen C. T. Storer H. L. (2017). What motivates men’s involvement in gender-based violence prevention? Latent class profiles and correlates in an international sample of men. Men and Masculinities, 20(3), 294–316. 10.1177/1097184X16634801

[bibr13-10778012251348425] Cooper L. B. Paluck E. L. Fletcher E. K. Ryan I. M. Branscombe N. R. Center T. J. (2013). Reducing gender-based violence. In Ryan M. K. Branscombe N. R. (Eds.), The Sage handbook of gender and psychology (pp. 359–378). Sage. 10.4135/9781446269930.n22

[bibr14-10778012251348425] Cooper K. R. Shumate M. (2012). Interorganizational collaboration explored through the bona fide network perspective. Management Communication Quarterly, 26(4), 623–654. 10.1177/0893318912462014

[bibr15-10778012251348425] Dworkin S. L. Barker G. (2019). Gender-transformative approaches to engaging men in reducing gender-based violence: A response to Brush & Miller’s “trouble in paradigm”. Violence Against Women, 25(14), 1657–1671. 10.1177/1077801219872555 31640533

[bibr16-10778012251348425] Elbers W. Arts B. J. (2011). Keeping body and soul together: Southern NGOs’ strategic responses to donor constraints. International Review of Administrative Sciences, 77(4), 713–732. 10.1177/0020852311419388

[bibr17-10778012251348425] Farnworth C. Vaarst M. (2012). Making it too simple? Researchers, recommendations, and NGOs in the Sundarbans, Indian West Bengal. Journal of Agriculture, Food Systems, and Community Development, 2(4), 137–146. 10.5304/jafscd.2012.024.001

[bibr18-10778012251348425] Gevers A. Jama-Shai N. Sikweyiya Y. (2013). Gender-based violence and the need for evidence-based primary prevention in South Africa: Perspectives. African Safety Promotion, 11(2), 14–20. https://hdl.handle.net/10520/EJC148763

[bibr19-10778012251348425] Gibbs A. Washington L. Abdelatif N. Chirwa E. Willan S. Shai N. Jewkes R. (2020). Stepping stones and creating futures intervention to prevent intimate partner violence among young people: Cluster randomized controlled trial. Journal of Adolescent Health, 66(3), 323–335. 10.1016/j.jadohealth.2019.10.004 31784410

[bibr20-10778012251348425] Gibson K. O’Donnell S. Rideout V. (2007). The project-funding regime: Complications for community organizations and their staff. Canadian Public Administration, 50(3), 411–435. 10.1111/j.1754-7121.2007.tb02135.x

[bibr21-10778012251348425] Girei E. (2023). Managerialisation, accountability and everyday resistance in the NGO sector: Whose interests matter? Critical Perspectives on Accounting, 92, Article 102418. 10.1016/j.cpa.2022.102418

[bibr22-10778012251348425] Ismail Z. (2019, March). *Advantages and value of funding NGOs in the global South. K4D helpdesk report*. https://gsdrc.org/publications/advantages-and-value-of-funding-ngos-in-the-global-south/

[bibr23-10778012251348425] Jewkes R. Nduna M. Levin J. Jama N. Dunkle K. Puren A. Duvvury N. (2008). Impact of stepping stones on incidence of HIV and HSV-2 and sexual behaviour in rural South Africa: Cluster randomised controlled trial. BMJ, 337(7666), 1461–1395. 10.1136/bmj.a506 PMC2505093 18687720

[bibr24-10778012251348425] Johannes L. (2019, September 4). #AmINext protests: South African women and students have had enough. *News24*. https://www.news24.com/life/in-pictures-south-african-women-and-children-have-had-enough-20190904

[bibr25-10778012251348425] Kieser J. (2023). A review of gender-based violence organisations in South Africa and their influences of decision-making [Master’s dissertation]. University of Cape Town. OpenUCT. http://hdl.handle.net/11427/39620

[bibr26-10778012251348425] Kim J. C. Watts C. H. Hargreaves J. R. Ndhlovu L. X. Phetla G. Morison L. A. Pronyk P. (2007). Understanding the impact of a microfinance-based intervention on women’s empowerment and the reduction of intimate partner violence in South Africa. American Journal of Public Health (1971), 97(10), 1794–1802. 10.2105/AJPH.2006.095521 PMC1994170 17761566

[bibr27-10778012251348425] Kirk L. Terry S. Lokuge K. Watterson J. L. (2017). Effectiveness of secondary and tertiary prevention for violence against women in low and low-middle income countries: A systematic review. BMC Public Health, 17(1), 622–622. 10.1186/s12889-017-4502-6 28676044 PMC5496243

[bibr28-10778012251348425] Mogoatlhe L. (2019, September 2). Uyinene Mrwetyana’s death shows South Africa’s femicide crisis*. Global Citizen*. https://www.globalcitizen.org/en/content/uyinene-mrwetyana-gender-violence-south-africa/

[bibr29-10778012251348425] Mueller-Hirth N. (2012). If you don’t count, you don’t count: Monitoring and evaluation in South African NGOs. Development and Change, 43(3), 649–670. 10.1111/j.1467-7660.2012.01776.x

[bibr30-10778012251348425] National Shelter Movement of South Africa. (2024). *Progressive sheltering: Strengthening South African Shelters. *National Indaba Report (No 4, 2024). Report. https://www.nsmsa.org.za/wp-content/uploads/2025/05/nsmsa-indaba-report-2024.pdf

[bibr31-10778012251348425] O’Brien J. E. Macy R. J. (2016). Culturally specific interventions for female survivors of gender-based violence. Aggression and Violent Behavior, 31, 48–60. 10.1016/j.avb.2016.07.005

[bibr32-10778012251348425] Olivier C. Hunt M. R. Ridde V. (2016). NGO–Researcher partnerships in global health research: Benefits, challenges, and approaches that promote success. Development in Practice, 26(4), 444–455. 10.1080/09614524.2016.1164122

[bibr33-10778012251348425] Ormel I. Salsberg J. Hunt M. Doucet A. Hinton L. Macaulay A. C. Law S. (2020). Key issues for participatory research in the design and implementation of humanitarian assistance: A scoping review. Global Health Action, 13(1), Article 1826730. 10.1080/16549716.2020.1826730 PMC7594848 33073736

[bibr34-10778012251348425] Rathgeber E. (2009). Gender and natural resource management: Livelihoods, mobility and interventions. Revue Canadienne D’études Du Développement, 28(3–4), 603–605. 10.1080/02255189.2009.9669232

[bibr35-10778012251348425] Republic of South Africa. (2020). *National strategic plan on gender-based violence and femicide*. https://www.gov.za/sites/default/files/images/GBV%20_booklet.pdf

[bibr36-10778012251348425] Richards J. (2008). The many approaches to organisational misbehaviour. Employee Relations, 30(6), 653–678. 10.1108/01425450810910046

[bibr37-10778012251348425] SaferSpaces. (2020). *Saferspaces Gazette 2019/20.* Civilian Secretariat for Police Service. https://www.saferspaces.org.za/uploads/files/VCP_Gazette_2019-20_-_Digital_Interactive.pdf

[bibr38-10778012251348425] Skevington S. M. Sovetkina E. C. Gillison F. B. (2012). A systematic review to quantitatively evaluate “Stepping Stones”: A participatory community-based HIV/AIDS prevention intervention. AIDS and Behavior, 17(3), 1025–1039. 10.1007/s10461-012-0327-6 23128978

[bibr39-10778012251348425] South African Police Service, Strategic Management. (2023, August 31). South African Police Service Annual Report 2022/2023. South African Police Service. https://www.saps.gov.za/about/stratframework/annual_report/2022_2023/Annual-Report-2022-23-final-draft-2023-10-12.pdf

